# The influence of social support on the physical exercise behavior of college students: The mediating role of self-efficacy

**DOI:** 10.3389/fpsyg.2022.1037518

**Published:** 2022-12-02

**Authors:** Yan Zhang, Hasibagen, Chang Zhang

**Affiliations:** ^1^School of Resource and Environmental Economy, Inner Mongolia University of Finance and Economics, Hohhot, China; ^2^School of Business, Central South University, Changsha, China

**Keywords:** social support, self-efficacy, structural equation model, physical exercise behavior, college students

## Abstract

**Objectives:**

To study the influencing factors on college students’ physical exercise behavior and the mediating relationship of self-efficacy based on the theory of social support and self-efficacy; to provide theoretical support and practical guidance for college students engaging independently in physical exercise.

**Methods:**

A total of 1,440 college students from six universities in the Inner Mongolia Autonomous Region of China were selected as the research objects, and three scales (Self-Efficacy Scale, Social Support Scale, and Physical Exercise Rating Scale) were used to construct a structural equation model.

**Results:**

(1) A comprehensive sports facility environment is conducive to college students’ physical activities and the emotional support of friends and family and the campus cultural atmosphere cannot be ignored. (2) Peer support has a direct impact on physical exercise behavior, family support and school support indirectly affect college students’ physical exercise behavior, based on the intermediary role of self-efficacy. (3) According to the total effect, social support was ranked as school support (0.444), peer support (0.312), and family support (0.145).

**Conclusion:**

Social support not only directly affects physical exercise behavior but also indirectly affects physical exercise behavior based on the mediating effect of self-efficacy.

## Introduction

In recent years, the physical health level of Chinese college students has been low, and, in the overall developmental quality of students, their physical quality has decreased (Li, 2007; Zhang et al., 2012). College students are in a critical period of their life growth transition. Stimulating college students to adhere to physical exercise and to have a healthy life will help increase the healthy population aspects of “Healthy China 2030” (Chen et al., 2017b). Colleges and universities pay more and more attention to the physical quality of students and often carry out a range of after-school training, competitions, and other activities. College students’ participation in sports is not only a current social requirement for the all-around development of college students but also an aspect of encouraging college students to form lifelong sports awareness and lifelong engagement in sports, an important social consideration (LaCount et al., 2022).

With the development of the concept of healthy China and of behavioral psychology, many scholars have conducted research on the characteristics of college students’ physical exercise behavior, on the internal and external influencing factors, and on physical exercise behavior mechanisms. Research has confirmed the applicability of self-efficacy theory and social support theory in the field of physical exercise (Schwarzer, 2000; Zhang et al., 2022). Social support theory holds that one of the important ways to encourage people to engage in physical exercise is to give them various supports when they are preparing for exercise (Kathleen et al., 2022). It refers to the function and quality of social relationships, such as either the perceived availability of help or support actually received (Warner et al., 2011). According to the health behavior model proposed by Mcleroy et al. (1988) human behavior is the result of the interaction of multiple systems. Social support exists on an interpersonal level and is at the near end of the health behavior model. Scholars have divided social support into separate categories according to the different environments of the research objects. Tang and Guo (2021) studied the impact of the use of sports and fitness apps on individual exercise behavior among sports and fitness app users over the age of 18. They separated social support into family support, friend support, and other support. Ji et al. (2022) studied the influence path of social support on the physical exercise behavior of college students in Beijing, at the level of social support, the authors only considered peer support and family support. Springer et al. (2006) studied the impact of social support on the physical activity of adolescent girls, mainly evaluating the role of friends and family in physical activity. The above studies have ignored the importance of environmental factors in the students’ main exercise facility and the cultural atmosphere of sports in the school. Some scholars have found that the lack of a campus sports culture is one of the fundamental problems among the factors restricting physical exercise in colleges and universities (Jing and Sun, 2006). A campus sports culture is the atmosphere of sports activities with the characteristics of the university. It is gradually formed by college students in sports teaching, fitness activities, sports competitions, sports facilities, and other activities. It is also an important part of the construction of a campus culture (Escamilla-Fajardo et al., 2020). Therefore, in this study, school support was added to the previous two categories of family and peer support, and social support was examined from three perspectives: school support, peer support, and family support. Family support refers to parents’ exercise behavior, parents’ financial support, and family fitness equipment (Dan and Yao, 2015). Peer support refers to the supportive behavior of relevant partners for individual physical exercise behaviors. School support refers to the shaping of students’ values by teachers, the guidance of physical exercise behavior, the level of sports facilities provided by schools, and the cultural atmosphere of physical exercise in schools.

American psychologist Bandura (1986) proposed the theory of self-efficacy, stating that individual behavior, cognition, and environment influence each other. Self-efficacy affects which activities people choose to engage in, the amount of effort they expend in these activities, the extent to which they persevere in the face of difficulties, and the cognitive evaluations and emotional reactions brought about by successes and failures (Bandura, 1977). It refers to the individual’s judgment of self as able to complete an activity and as having the confidence and assurance to complete it (François, 2004). Exercise self-efficacy is an individual’s perception of his or her extent of feeling capable to perform physical exercise to achieve a certain outcome in the future (Warner et al., 2011). The sense of self-efficacy is of great significance in the initial process of people’s participation in physical exercise. The higher the self-efficacy, the better the performance in the initial stage of physical exercises. This is because self-efficacy can help participants overcome some obstacles to physical exercise, such as overcoming learning pressure, persisting in physical exercise, and continuing to engage in physical exercise even if they do not achieve the desired motivational effect. A good sense of self-efficacy can increase college students’ confidence and enthusiasm for taking an active part in physical exercise (Motl et al., 2007).

A substantial body of research has studied college students’ physical exercise behavior. However, few studies have investigated the influencing factors of college students’ group physical exercise based on the combination of social support theory and self-efficacy theory. Research has largely ignored the crucial significance of the construction of sports culture in school support. Therefore, this paper attempts to start from the social support theory and the self-efficacy theory, and deeply study the path relationship between the two in college students’ physical exercise behavior. This study constitutes a significant advance in the promotion of college students’ independence and adherence to physical exercise. The aim of this study is to provide a theoretical basis and practical guidance for the promotion of college students’ physical health.

## Research hypothesis

### The relationship between social support and physical exercise behavior

Social support is a key variable that affects young people’s physical exercise activities (Courneya et al., 2000; Lippke, 2004). Adequate social support is an effective way to foster young people’s participation in physical exercise (Ren et al., 2021). Dong et al. (2018) found that social support can buffer the negative effects of physical exercise discomfort on the body and mind while enhancing the sense of pleasure and satisfaction derived from exercise. Carron et al. (1996) found that family support and support of significant others as important factors for exercise adherence behavior. College students who perceive social support can gain the internal motivation to take an active part in physical exercise. Accordingly, the following hypotheses are proposed in this study:

*H1*: Peer support has a significant positive effect on college students’ physical exercise behavior.

*H2*: Family support has a significant positive effect on college students’ physical exercise behavior.

*H3*: School support has a significant positive effect on college students’ physical exercise behavior.

### The relationship between social support and self-efficacy

Social support and self-efficacy are not independent of each other. Social cognitive theory (Bandura, 1977) suggests interactions of self-efficacy and social support: Self-efficacy could moderate the effects of social support on physical exercise in a synergistic manner, in that individuals with higher self-efficacy profit more from support because they are more likely to translate support into exercise. Studies have shown that a satisfactory social environment and interpersonal support are conducive to improving individual confidence in participating in sports activities (Kang et al., 2019). Social support has an impact on the individual’s understanding and is conducive to the generation of self-efficacy, thereby affecting the individual’s behavior (Chen and She, 2019). Therefore, the following hypotheses are proposed:

*H4*: School support has a significant positive effect on self-efficacy.

*H5*: Family support has a significant positive effect on self-efficacy.

*H6*: Peer support has a significant positive effect on self-efficacy.

### The relationship between self-efficacy and physical exercise behavior

A large number of studies have confirmed the inseparable relationship between self-efficacy and physical exercise behavior. Self-efficacy is usually considered the most common intermediary variable affecting sports activities (Dong and Mao, 2018). Klompstra et al. (2018) found that in addition to a high level of motivation to be physically active, it is important that patients with heart failure have a high degree of self-efficacy. Cheng (2019) found that self-efficacy plays a mediated role between friend support and physical activity. Ray and Henry (2011) examined the relationship between self-efficacy and physical activity in children aged 10–14 years with congenital heart disease, the results showed that self-efficacy scores were correlated with physical activity participation. Therefore, this hypothesis is proposed:

*H7*: Self-efficacy has a significant positive impact on physical exercise behavior.

## Objectives and methods

### Objects

College students from six universities in Hohhot, Inner Mongolia Autonomous Region, were selected as the research subjects. A questionnaire was completed for distribution on-site and online. The survey was conducted in two stages. In the first stage, a total of 145 students from three classes at the Inner Mongolia University of Finance and Economics were selected for the survey; 10 invalid questionnaires were excluded. The 135 valid questionnaires obtained in the first stage were used for reliability and validity tests. On successful completion of the tests, the second phase of the formal investigation began. Each school distributed 250 questionnaires, a total of 1,500. When the questionnaire was distributed on-site, students were randomly selected. The online questionnaires were distributed to the students with the help of the head teacher. A total of 1,440 valid questionnaires were collected, 46% of the respondents were male and 54% were female. The responses to the questions used a 5-level Likert scale in which 1–5 points were assigned according to “strongly agree,” “agree,” “neutral,” “disagree,” and “strongly disagree,” respectively. When analyzing the physical exercise behavior of college students, the duration, intensity, and frequency of independent physical exercise in college were considered, and a score of 1–5 was assigned.

### Methods

#### Physical exercise rating scale

The Physical Exercise Rating Scale, revised by the Chinese scholar Liang (1994) has become the main index for measuring individual physical exercise behavior. It is used to detect the intensity, duration, and frequency of exercise.

#### Exercise self-efficacy scale

Four questions suitable for the actual situation of college students were adapted from the exercise self-efficacy scale revised by Kroll et al. (2007). For example, “I can be involved in physical exercises without being asked by teachers,” “I will continue to exercise during winter and summer vacations.”

#### Social support scale

Social support was measured by referring to the Social Support Scale compiled by Chen et al. (2016), which was divided into three dimensions: family, friends, and others. The scale was modified, questions were added, and the questions were divided into three dimensions: peer support, school support, and family support. Questions setting of peers and families can be divided into two aspects: emotional support (such as “Your friend comforts you when you experience negative emotions during exercise”) and examples of behavior (such as “Your parents exercise, most of your friends exercise often.”). School support can be divided into three aspects: subject support (such as teachers’ encouragement) and quality of sports facilities (such as “The school has perfect places for physical exercise.”), and sports culture atmosphere (such as “The school often holds large-scale sports activities, and many people participate”). Table 1 shows the scales and questionnaire questions.

**Table 1:** Questionnaire setting of latent and observed variables.

Latent variable type	Latent variables	Observed variables	Symbols
Endogenous latent variables	Self-efficacy (U1)	Even when my partner cannot accompany me, I will engage in physical exercise	U11
		I engage in exercise without the teacher’s request	U12
		I will continue to exercise during the winter and summer vacations	U13
		I can overcome obstacles and challenges in physical exercise	U14
	Exercise behavior (U5)	Duration of exercise	U51
		Exercise intensity	U52
		Exercise frequency	U53
Exogenous latent variables	Peer support (U2)	Most of your friends take part in sports	U21
		Your friends often encourage you to take part in physical exercise	U22
		Your friends are willing to adjust their schedule to accompany you when you participate in exercise	U23
		Your friend comforts you when you experience negative emotions during exercise	U24
	School support (U3)	Your school often holds sports activities such as enjoyable games	U31
		The basketball court, badminton hall, and other sports places in the school are always crowded	U32
		The sports grounds and facilities in your school meet your needs for physical exercise	U33
		The PE teacher/head teacher often encourages you to participate in physical exercise	U34
		There are many participants in the sports activities held by your school	U35
	Family support (U4)	Your parents encourage you to take part in physical exercise	U41
		Your parents provide financial support for your physical exercise	U42
		Your parents often exercise	U43
		There are many items of sports equipment in your home that meet your need for exercise	U44

### Statistical analysis

In the selection of research methods, the traditional linear analysis and correlation analysis were rejected in favor of the structural equation model. The model is suitable for exploring the influence of multiple variables on dependent variables and can analyze the relationship between latent variables as well as between observed variables and latent variables (Aderson and Gerbing, 1988). Therefore, this method is sufficiently thorough to accurately identify the multiple factors affecting college students’ physical exercise behavior and the path relationship (Yu et al., 2021). The model includes a measurement model and a structural model. In addition, some important formulas will also be introduced in this section.

#### Measurement model

The measurement model consists of latent variables and observed variables. It comprises two equations used to express the relationship between exogenous observed variables (U21–U44) and exogenous latent variables (U2, U3, and U4), and the relationship between endogenous observed variables (U11–U14, U51–U53) and endogenous latent variables (U1, U5). The specific form of the model is as follows:


(1) $$\begin{array}{c} X\\ \left(13\times 1\right) \end{array}=\begin{array}{c} \Lambda x\\ \left(13\times 3\right) \end{array}\begin{array}{c} \xi \\ \left(3\times 1\right) \end{array}+\begin{array}{c} \delta \\ \left(13\times 1\right) \end{array}$$

(2) $$\begin{array}{c} Y\\ \left(7\times 1\right) \end{array}=\begin{array}{c} \Lambda Y\\ \left(7\times 2\right) \end{array}\begin{array}{c} \eta \\ \left(2\times 1\right) \end{array}+\begin{array}{c} \varepsilon \\ \left(7\times 1\right) \end{array}$$

In the formulas, $\begin{array}{c} X\\ \left(13\times 1\right) \end{array}$ is the vector of exogenous observed variables; $\begin{array}{c} Y\\ \left(7\times 1\right) \end{array}$ is the vector of endogenous observed variables; $\begin{array}{c} \Lambda x\\ \left(13\times 3\right) \end{array}$ is the factor load matrix of exogenous observed variables on exogenous latent variables; $\begin{array}{c} \Lambda Y\\ \left(7\times 2\right) \end{array}$ is the factor load matrix of endogenous observed variables on endogenous latent variables; $\begin{array}{c} \xi \\ \left(3\times 1\right) \end{array}$ is the vector composed of exogenous latent variables$\begin{array}{c} \eta \\ \left(2\times 1\right) \end{array}$ is the vector of endogenous latent variables; $\begin{array}{c} \delta \\ \left(13\times 1\right) \end{array}$ is the errors term vector of exogenous observed variables; and $\begin{array}{c} \varepsilon \\ \left(7\times 1\right) \end{array}$ is the errors term vector of endogenous observed variables.

#### Structural model

The structural model is used to explain the relationship between exogenous latent variables (U2, U3, and U4) and endogenous latent variables (U1 and U5). The formula is as follows:


(3) $$\begin{array}{c} \eta \\ \left(2\times 1\right) \end{array}=\begin{array}{c} B\\ \left(2\times 2\right) \end{array}\begin{array}{c} \eta \\ \left(2\times 1\right) \end{array}+\begin{array}{c} \Gamma \\ \left(2\times 3\right) \end{array}\begin{array}{c} \xi \\ \left(3\times 1\right) \end{array}+\begin{array}{c} \psi \\ \left(2\times 1\right) \end{array}$$

$\begin{array}{c} B\\ \left(2\times 2\right) \end{array}$ is the coefficient matrix of endogenous latent variables, describing the mutual influence between endogenous latent variables; $\begin{array}{c} \Gamma \\ \left(2\times 3\right) \end{array}$ is the coefficient matrix of exogenous latent variables, describing the influence of exogenous latent variables on endogenous latent variables; and $\begin{array}{c} \psi \\ \left(2\times 1\right) \end{array}$ is the residual vector, reflecting the part that cannot be explained in the equation.

#### Calculation of other values

Topic reliability (represented by squared multiple correlations, or SMC), composite reliability (CR), and average variance extracted (AVE) values were calculated as follows with factor loading values, λ_i_, where i refers to different observed variables (Fornell and Larcker, 1981):


(4) $$\mathrm{SMC}=\lambda_{i}{}^{2}$$

(5) $$\mathrm{CR}=\frac{{\left(\sum \lambda_{i}\right)}^{2}}{\left[{\left(\sum \lambda_{i}\right)}^{2}+\sum \left(1-\lambda_{i}{}^{2}\right)\right]}$$

(6) $$\text{AVE}=\frac{\left(\sum \lambda_{i}{}^{2}\right)}{\left[\left(\sum \lambda_{i}{}^{2}\right)+\sum \left(1-\lambda_{i}{}^{2}\right)\right]}$$

## Results

### Results of confirmatory factor analysis

Confirmatory factor analysis (CFA) was used to verify constructive validity. When the data show good constructive validity, they must have convergent validity and discriminant validity. Independent CFA was conducted on self-efficacy, physical exercise behavior, peer support, school support, and family support, respectively. The measurement models of self-efficacy, peer support, school support, and family support required adjustment and correction. All indicators with factor loading values less than 0.5 (U12, U41, U35, and U21) were deleted to improve the model fit and constructive validity. Tables 2, 3 show the results of CFA and discriminant validity analysis after exclusion.

**Table 2:** Confirmatory factor analysis.

		Factor loadings	Topic reliability	Composite reliability	Convergent validity
		Std.	SMC	CR	AVE
U1	U11	0.632	0.399	0.682	0.417
	U13	0.608	0.370		
	U14	0.695	0.483		
U2	U22	0.839	0.704	0.825	0.613
	U23	0.825	0.681		
	U24	0.673	0.453		
U3	U31	0.636	0.404	0.815	0.529
	U32	0.774	0.599		
	U33	0.846	0.716		
	U34	0.629	0.396		
U4	U42	0.544	0.296	0.646	0.380
	U43	0.637	0.406		
	U44	0.662	0.438		
U5	U51	0.848	0.719	0.777	0.540
	U52	0.674	0.454		
	U53	0.669	0.448		

Std. standardized estimate; CR, composite reliability; AVE, average variance extracted; SMC, squared multiple correlations.

**Table 3:** Discriminant validity test.

	Convergent validity	Pearson correlation and discriminant validity				
	AVE	U1	U2	U3	U4	U5
U1	0.417	**0.646**				
U2	0.613	0.189	**0.783**			
U3	0.529	0.143	0.267	**0.727**		
U4	0.380	0.250	0.293	0.134	**0.616**	
U5	0.540	0.273	0.363	0.382	0.286	**0.735**

Boldface numbers are square-root AVE values.

In Table 2, the factor loading values of each observed variable, apart from U42, is greater than 0.6, and the factor loading value of U42 is between 0.5 and 0.6. The CR values of U2, U3, and U5 are greater than 0.7, and those values of U1 and U4 are close to 0.7, indicating that the reliability of model construction is appropriate. The AVE values of U2, U3, and U5 are all greater than 0.5, those values of U1 and U4 are between 0.36 and 0.5, within the permitted range (Purnomo, 2017), indicating that the convergent validity of the model is good. Because Chin (1998) suggested that the standardized factor loadings should ideally be greater than the threshold of 0.7, with 0.6 or more being the acceptable range, and the square of the factor loadings is the AVE value, the AVE value is acceptable as long as it is greater than 0.36. And Fornell and Larcker (1981) said that if AVE is less than 0.5, but the CR value is higher than 0.6, the convergent validity of the construct is still adequate. The square roots of the diagonal AVE values in the Table 3 (indicated in bold) are larger than the correlation coefficients between the dimensions shown below the diagonal values. Therefore, there is a significant difference in validity among the dimensions.

### Model verification and modification

After removing the four observed variables U12, U41, U35, and U21 with factor loading values less than 0.5, the measurement model is rebuilt as follows:


(7) $$\begin{array}{c} X\\ \left(10\times 1\right) \end{array}=\begin{array}{c} \Lambda x\\ \left(10\times 3\right) \end{array}\begin{array}{c} \xi \\ \left(3\times 1\right) \end{array}+\begin{array}{c} \delta \\ \left(10\times 1\right) \end{array}$$

(8) $$\begin{array}{c} Y\\ \left(6\times 1\right) \end{array}=\begin{array}{c} \Lambda Y\\ \left(6\times 2\right) \end{array}\begin{array}{c} \eta \\ \left(2\times 1\right) \end{array}+\begin{array}{c} \varepsilon \\ \left(6\times 1\right) \end{array}$$

According to the newly constructed measurement model (Equations 7 and 8) and combined with Equation 3, the structural equation model of the influencing factors of college students’ physical exercise behavior is fitted and tested. Amos 26.0 was used to analyze the path of the data obtained from the 1,440 valid questionnaires. Table 4 shows the fit index of the modified optimal model. The main purpose of the model fitness test is to verify the degree of the fit between the theoretical model and the actual data of the structural equation model. A series of fit indices were tested, including relative/normed chi-square statistic (CMIN/DF), root mean square error of approximation (RMSEA), the goodness-of-fit statistic (GFI), the adjusted goodness-of-fit statistic (AGFI), and so on. All fitness indexes in the model are in line with the standard, indicating that theoretical data and actual model fit better.

**Table 4:** Index table of model fitness.

Model fitting index	CMIN/DF	RMSEA	GFI	AGFI	NFI	CFI	IFI
Standard	1–3	<0.08	>0.9	>0.9	>0.9	>0.9	>0.9
Model fitting degree	2.876	0.079	0.910	0.903	0.901	0.908	0.909

CMIN/DF, Discrepancy divided by degree of freedom; RMSEA, Root mean square error of approximation; GFI, Goodness-of-fit index; AGFI, Adjusted goodness-of-fit index; NFI, Normed fit index; CFI, Comparative fit index; IFI, incremental fit index.

The maximum likelihood estimation method is used to estimate the path coefficients. First, the insignificant path is modified and deleted. Second, the modification index (MI) of the model is considered, whereby the correction index is used to relax the restriction of some paths, and the transition to the saturation model makes the path effect more significant. After the modification of the model, five of the seven original hypotheses are valid, namely, UI ← U4 (*H5*), U5 ← U2 (*H1*), U5 ← U3 (*H3*), U5 ← U1 (*H7*), and U1 ← U3 (*H4*), as Table 5 shows.

**Table 5:** Standardized path coefficients and hypothesis testing results.

Parameter path	S.E.	C.R.	*p*-value	Estimate	Validation results
UI ← U4	0.067	7.093	<0.001^***^	0.476	Supported
U5 ← U2	0.104	4.118	<0.001^***^	0.430	Supported
U5 ← U3	0.084	5.949	<0.001^***^	0.501	Supported
U5 ← U1	0.092	4.037	<0.001^***^	0.371	Supported
U1 ← U3	0.055	3.376	<0.001^***^	0.186	Supported

*** A statistical significance of *p* < 0.001. S.E., standard error; C.R., critical ratio.

### Model interpretation

**Figure 1:**
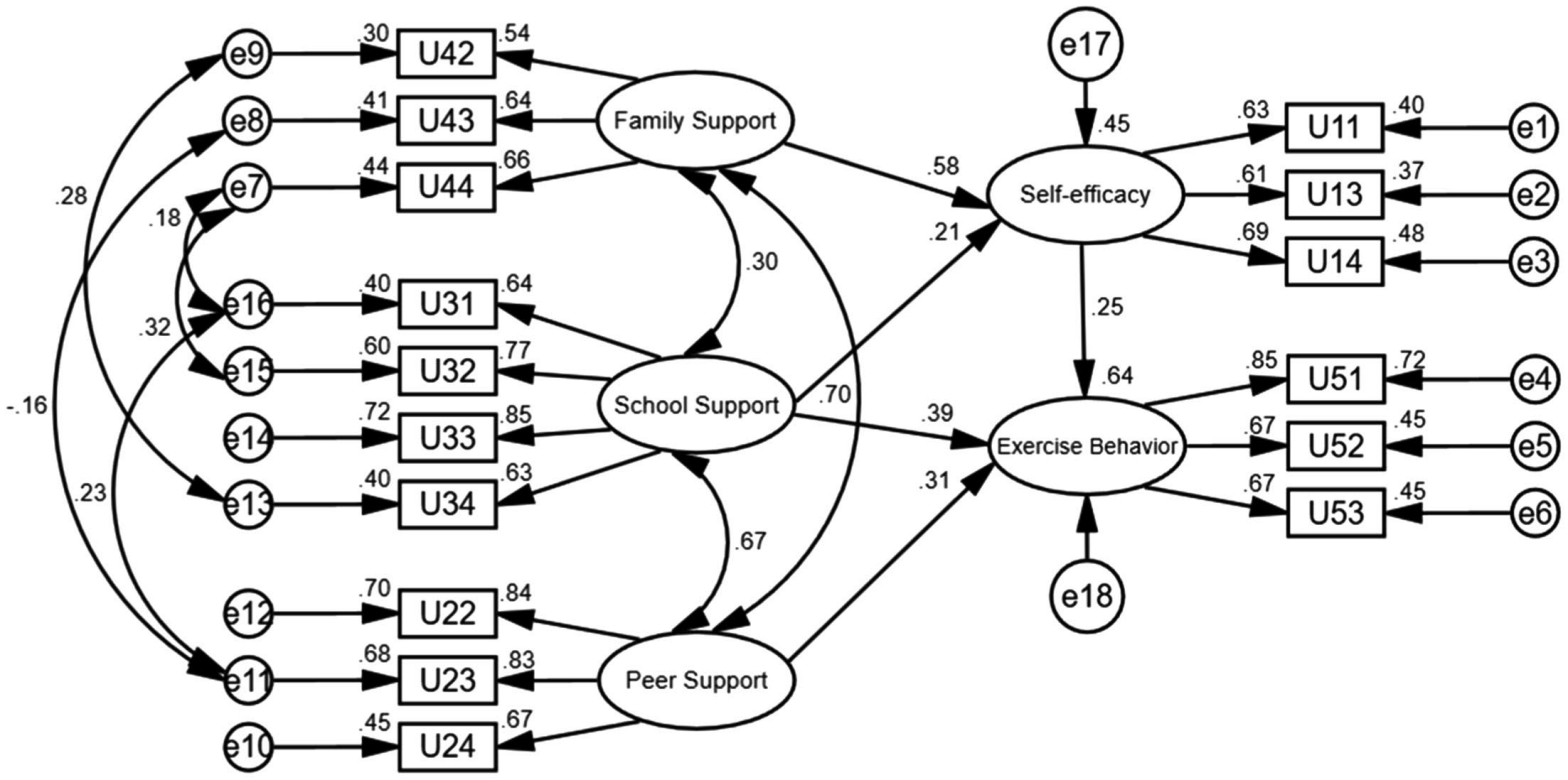
Path analysis of structural equation model.

Figure 1 shows the path coefficient obtained in the empirical analysis. Each observed variable reflects its corresponding latent variable. The measurement of influencing factors of exogenous latent variables shows that U44 (sports equipment at home can meet your need for exercise), at 0.66, has the largest impact on U4 (family support), indicating that the availability of sports equipment at home can best satisfy college students’ needs for family support. Among U3 (school support) measurement variables, U33 (school sports venues and facilities can meet your exercise needs) has the largest impact, 0.85, and school venues and facilities best reflect college students’ needs for school support. Among the measurement variables of U2 (peer support), U22 (friends’ encouragement) and U23 (friends’ willingness to spend time to accompany you when you participate in physical exercise) have the greatest impact, 0.84 and 0.83, respectively. Friends’ encouragement and companionship best reflect college students’ need for peer support. Results for other observed variables are all above 0.6, showing that the physical exercise behavior of college students needs the support of family, school, and peers. They also show that sports facilities are essential, family and peer emotional support are effective, and the school sports culture plays a role in promoting college students’ physical exercise behavior.

Combining Table 5 and Figure 1, the relationship between social support (family support, school support, peer support), self-efficacy, and physical exercise behavior is as follows: (1) The influence of family support on physical exercise behavior is only achieved through the intermediary role of self-efficacy (*H5* and *H7* are supported). (2)The impact of school support on physical exercise behavior is not only achieved through the intermediary effect of self-efficacy but also has a direct influence (*H3* and *H4* are supported). (3) Peer support has a direct impact on physical exercise behavior (*H1* is supported). The total effect is calculated by summing up the direct and indirect effects of social support on physical exercise behavior (Jodie and Ullman, 2006). The significant positive effects of school support, family support, and peer support on college students’ physical exercise behaviors were ranked as follows: school support (0.214 × 0.252 + 0.39 = 0.444) > peer support (0.312) > family support (0.576 × 0.252 = 0.145). School support had the largest effect, followed by peer and family support.

## Discussion

### The influence of school support on college students’ physical exercise behavior and the mediating effect of self-efficacy

The above results show that college students’ physical exercise behavior is most affected by school support. For students, their school or university is the most important place in their daily lives and the most likely place for students to use exercise facilities. Therefore, sports behavior activities are strongly influenced by various aspects of the school or university, such as the school’s advocacy of students’ values and the formation of ideological consciousness. Good exercise conditions and more exercise opportunities in schools can increase the number of students participating in physical exercise (Dong and Mao, 2018). Among the observed variables supported by the school, venues, and facilities are important for college students’ physical exercise. An ideal environment of sports facilities enhances students’ enjoyment of sports. The construction of a campus sports culture is also of great significance. The foundation of sports education lies in sports culture. In the absence of a sports culture, sports cannot be developed (Jing and Sun, 2006). Schools should play a leading role in promoting students’ physical exercise. They should increase their investment in sports facilities, hold more sports-related activities, strengthen the construction of a sports culture, and encourage students’ active participation in sports activities.

The influence of the school’s physical environment on students’ active participation in exercise can be realized through the intermediary of self-efficacy (Sun, 2019). This is further verified in this paper. In addition to directly influencing college students’ physical exercise behavior, school support will also indirectly affect college students’ physical exercise behavior through self-efficacy. The higher the sense of self-efficacy, the higher the enthusiasm to take part in physical exercise.

### The influence of peer support on physical exercise behavior of college students and the mediating effect of self-efficacy

Compared with school support, peer support had the second most significant effect on college students’ physical exercise behavior. Peer relationships play a major role in motivating college students to engage in physical exercise (Bull et al., 2020). Yang (2016) research shows that when college students feel the support of enough friends, they will participate more confidently in physical exercise and will try to overcome any obstacles to physical exercise. Haidar et al. (2019) also used college students as research objects to explore the relationship between peer support and physical exercise. Every unit of peer support increases the probability of college students participating in physical activities by at least 1.15 times. Friends’ encouragement and support are essential in promoting college students’ physical exercise behavior. The findings of this paper are consistent with the research findings of the above scholars that peer support has a significant positive impact on college students’ physical exercise behavior.

Peer support, self-efficacy, and physical exercise behavior are correlated to some extent (Yang, 2016). Existing research conclusions are inconsistent as to whether peer support directly affects people’s physical activities. Chen et al. (2017a) investigated students from Fuzhou, Fujian Province, and found that peer support did not directly affect physical activity, but indirectly affected physical activity based on the intermediary relationship of self-efficacy. Duncan et al. (2007) found that peer support directly affected adolescents’ sports activities. The structural equation model constructed in this paper leads to the conclusion that peer support can only directly affect college students’ physical activities, but that it has nothing to do with self-efficacy. This may be related to the overall model construction, sample selection, and the number of samples. Therefore, the single influence relationship between the three aspects needs further research.

### The influence of family support on college students’ physical exercise behavior and the mediation of self-efficacy

The influence of family support on college students’ physical exercise behavior is relatively weak. Family is the primary environment where children develop habits. We should focus on the influence of family factors on college students’ physical exercise, and implement educational means and methods according to the specific situation of each family (Peng, 2003). Parents are the first teachers of their children, and they have an influence on their children. However, in college, students become more independent due to the distance between them and their parents, and the impact on their families becomes weaker. Therefore, family support has a secondary impact on college students’ physical exercise when compared with school support and peer support.

Self-efficacy is the bridge between family support and physical exercise behavior. Family support only indirectly affects college students’ physical exercise behavior through the intermediary effect of self-efficacy. For example, the availability of sports equipment at home and the physical activity behavior of parents will affect the physical exercise of students, who are not directly affected but are indirectly affected through the intermediary of self-efficacy. The family sports atmosphere greatly increases students’ confidence in physical exercise and encourages them to participate in and persist in physical exercise. Parents should set an example for their children, working together to create a good atmosphere and environment for physical exercise.

## Research conclusions and limitations

### Research conclusions

Based on the survey data of college students in six colleges and universities in Hohhot, Inner Mongolia, and the theory of social support and self-efficacy, a structural equation model was built to study the relationship between external support, self-efficacy, and college students’ physical exercise behavior. The research conclusions show the following:

1. The analysis of the relationship between observed variables and latent variables shows that a comprehensive sports facility environment is conducive to college students’ physical activities and the emotional support of friends and family and the campus cultural atmosphere cannot be ignored.
2. The structural equation model of social support, self-efficacy, and college students’ physical exercise behavior had a good overall fit, confirming that college students’ physical exercise behavior is affected by the four dimensions of school support, family support, peer support, and self-efficacy. Among them, self-efficacy has only a mediating role in family support and school support, while peer support directly affects physical exercise behavior.
3. The effects of social support on the physical exercise of college students were as follows: school support (0.444), peer support (0.312), and family support (0.145). School support has the greatest influence on physical exercise behavior, followed by peer and family support.

### Limitations

1. Due to the impact of the COVID-19 epidemic, it was not possible to investigate and examine the physical exercise behavior data of college students from multiple schools. The research objects are students from only six universities in Inner Mongolia. The lack of samples may result in a lack of accuracy in the research conclusions.
2. The innovation and discovery aspects of this research lie in identifying the importance of the sports campus cultural atmosphere. However, the topics on the scales are limited, and there is no targeted research and analysis on sports cultural atmosphere. There is a need to improve the topic setting and analysis of the scale in future research.

## Data availability statement

The raw data supporting the conclusions of this article will be made available by the authors, without undue reservation.

## Author contributions

YZ was responsible for the design of the study and essay writing. H was responsible for revising the study. CZ was responsible for collecting the data. All authors contributed to the article and approved the submitted version.

## Funding

This study was supported by the Inner Mongolia Natural Science Foundation project “A study of socio-spatial differentiation and ethnic intermingling in multi-ethnic cities from the perspective of spatial behavior in time” (No. 2021MS04006) and Inner Mongolia Science and Technology Department project “Inner Mongolia tourism big data platform construction and key technology innovation” (No. 2020GG0105).

## Conflict of interest

The authors declare that the research was conducted in the absence of any commercial or financial relationships that could be construed as a potential conflict of interest.

## Publisher’s note

All claims expressed in this article are solely those of the authors and do not necessarily represent those of their affiliated organizations, or those of the publisher, the editors and the reviewers. Any product that may be evaluated in this article, or claim that may be made by its manufacturer, is not guaranteed or endorsed by the publisher.
